# Sarcoïdose hypophysaire mimant un adénome hypophysaire: à propos d'un cas et revue de la littérature

**DOI:** 10.11604/pamj.2019.33.92.17881

**Published:** 2019-06-07

**Authors:** Fahd Derkaoui Hassani, Mustapha Fadli, Najia El Abbadi

**Affiliations:** 1 Service de Neurochirurgie, Hôpital Universitaire International Cheikh Zaid, Rabat, Maroc; 2 Université Mohammed V de Rabat, Rabat, Maroc; 3 Université Internationale Abulcasis des Sciences de la Santé, Rabat, Maroc

**Keywords:** Sarcoïdose, neurosarcoïdose, hypophyse, hypothalamus, selle turcique, endoscopie, Sarcoidosis, neurosarcoidosis, pituitary gland, hypothalamus, sella turcica, endoscopy

## Abstract

L'atteinte isolée de l'axe hypothalamo-hypophysaire dans la sarcoïdose est rare. Seulement quelques cas sont rapportés sur la littérature. Nous rapportons l'observation clinique d'une patiente âgée de 50 ans, opérée il y a 4 ans pour goitre avec thyroïdectomie totale sous traitement substitutif. Elle se plaint de céphalées chroniques depuis 6 mois devenues récemment rebelles concomitant avec la survenue d'une baisse de l'acuité visuelle. L'examen clinique retrouve un syndrome opto-chiasmatique avec altération importante du champ visuel. Elle présente aussi une paralysie du nerf occulo-moteur commun à droite avec ptôsis. L'IRM cérébrale a objectivé un processus sellaire de signal tissulaire avec prise de contraste hétérogène et qui s'étend à la tige pituitaire avec épaississement de celle-ci. Le bilan hormonal pré opératoire a retrouvé une légère insuffisance thyréotrope. La patiente a bénéficié d'une exérèse tumorale large par voie transnasale trans-sphénoidale endoscopique d'une tumeur de consistance fibreuse et peu hémorragique. L'évolution post opératoire était marquée par la survenue d'un diabète insipide et d'une fistule de LCR au 2^ème^ jour post opératoire. L'évolution était bonne sous traitement médical avec drainage spinal. Le bilan général n'a pas retrouvé une autre localisation de la sarcoïdose notamment une tomodensitométrie thoraco abdominale et échographie cardiaque sans particularités. Le dosage de l'enzyme de conversion est revenu normal. La patiente a bénéficié d'une corticothérapie pour le traitement de la maladie systémique. La neuro sarcoïdose est un critère de mauvais pronostic chez un patient atteint d'une sarcoïdose. L'atteinte hypothalamo-hypophysaire est rare avec des complications plus fréquentes que les autres atteintes neurologiques et systémiques nécessitant une prise en charge multidisciplinaire à long terme.

## Abstract (en)

Isolated involvement of the hypothalamic-pituitary axis in patients with sarcoidosis is rare. Only a few cases have been reported in the literature. We report the clinical case of a 50-years old female patient who had undergone total thyroidectomy followed by replacement therapy because of goitre 4 years before. She complained of chronic headaches persisting for 6 months and resistant to all appropriate therapies, with concomitant decrease in visual acuity. Clinical examination showed optochiasmatic syndrome with extensive visual field defect and common oculomotor nerve palsy in the right eye with ptosis. Brain MRI objectified pathological process to the sellar region with heterogeneous tissue signal intensity extending to the pituitary stalk with thickening in the latter. Preoperative hormonal assessment showed mild thyrotropic deficiency. The patient underwent wide transnasal endoscopic transsphenoidal resection of fibrous and little bleeding tumor. Postoperative outcome was marked by the occurrence of diabetes insipidus and CSF fistula two days after surgery. Patient’s outcome was good under medical treatment with spinal drainage. Thoracoabdominal CT scan and cardiac ultrasound were performed which showed no other site of occurrence of sarcoidosis. Dose of converting enzyme was normal. The patient received corticosteroid therapy for the treatment of systemic disease. Neurosarcoidosis is a criterion of poor prognosis in a patient with sarcoidosis. Hypothalamopituitary involvement is rare resulting in complications which are more frequent than those of other neurological and systemic disorders. This requires multidisciplinary long term management.

## Introduction

L'atteinte isolée de l'axe hypothalamo-hypophysaire dans la sarcoïdose est rare [1]. Seulement quelques cas sont rapportés sur la littérature. Nous rapportons un cas de sarcoïdose hypothalamo-hypophysaire avec revue de la littérature.

## Patient et observation

**Figure 1:**
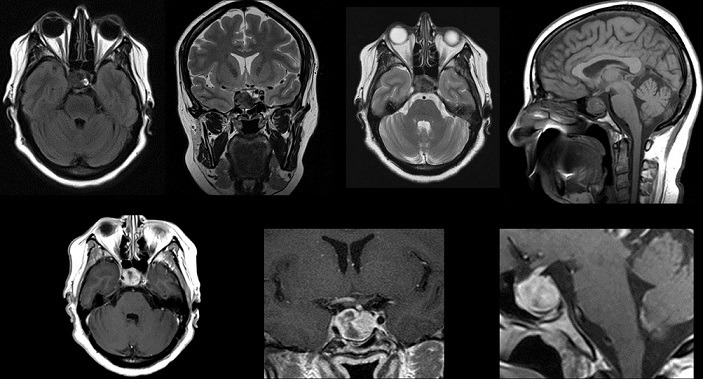
IRM pré opératoire d´un processus sellaire, lésion sellaire iso intense en séquences T1 avec un rehaussement hétérogène après injection de gadolinium avec extension au sinus caverneux à droite; on note aussi le rehaussement et l´épaississement de la tige pituitaire après injection de produit de contraste; les voies optiques sont libres

Nous rapportons l'observation clinique d'une patiente âgée de 50 ans, déjà opérée il y a 4 ans pour un goitre avec thyroïdectomie totale. Elle se plaint de céphalées chroniques depuis 6 mois devenues récemment rebelles concomitant avec la survenue d'une baisse de l'acuité visuelle. L'examen clinique retrouve un syndrome opto-chiasmatique avec altération importante du champ visuel, acuité visuelle à 8/10 en ODG et un fond d'oeil normal. Elle présente aussi une paralysie du nerf occulo-moteur commun à droite avec ptôsis. L'IRM cérébrale a objectivé un processus sellaire de signal tissulaire avec prise de contraste hétérogène et qui s'étend à la tige pituitaire avec épaississement de celle-ci (Figure 1). Le bilan hormonal pré opératoire a retrouvé une légère insuffisance thyréotrope (taux de triodothyronine « T3L » à 1,75 pg/ml) corrigée par l'administration d'hormones thyroïdiennes de synthèse. Les taux des autres lignées hypophysaires sont: cortisol de 8h du matin à 22,2 ug/dl, cortisol de 16h à 11,1, prolactine à 12,88 ng/ml, FSH à 12,95 mUI/ml, LH à 4,91 mUI/ml, TSHus à 0,44 uUI/ml, Thyroxine libre (T4L) à 1,03. La patiente a bénéficié d'une exérèse tumorale par voie transnasale trans-sphénoidale endoscopique avec ablation partielle d'une tumeur de consistance dure, fibreuse et peu hémorragique. L'évolution post opératoire était marquée par la survenue d'un diabète insipide et d'une fistule de LCR au 2^ème^ jour post opératoire. L'évolution était bonne sous traitement médical avec drainage spinal. Le bilan général n'a pas retrouvé une autre localisation de la sarcoïdose notamment une tomodensitométrie thoraco-abdominale et échographie cardiaque sans particularités. Le dosage de l'enzyme de conversion est revenu normal. La patiente a bénéficié d'une corticothérapie pour le traitement de la maladie systémique.

## Discussion

La sarcoïdose est une maladie systémique chronique d'étiologie inconnue qui se caractérise par la présence d'un granulome épithéloïde sans nécrose caséeuse [2]. Son incidence diffère selon la zone géographique et l'ethnie [3]. Elle est de 5-40 cas/100 000 habitants au niveau de l'Europe du nord. Au Japon, cette incidence est de l'ordre de 1 à 2 cas / 100 000 habitants. Aux états unis, cette incidence diffère entre les afro-américains et caucasiens (35,5 vs 10,5 cas / 100 000 habitants) [3]. La neurosarcoïdose est retrouvée chez 5 à 26% des patients atteints de sarcoïdose [4]. Au Japon, 6,8% des patients atteints de sarcoïdose présentent une atteinte du système nerveux central [5]. L'atteinte neurologique est retrouvée dans 25% des cas sur une série d'autopsie de patients atteints de sarcoïdose [3]. Enfin, la neurosarcoïdose peut être la première manifestation de cette granulomatose [2]. L'atteinte hypothalamo-hypophysaire isolée est rare (Tableau 1). Cette localisation représente 0,5% des cas de sarcoïdose et 1% des lésions de la selle turcique [2, 6]. Les principales manifestations hypothamalo-hypophysaires sont les troubles endocriniens et l'hypercalcémie [6-8]. Les manifestations ophtalmologiques d'une atteinte hypothalamo-hypophysaire sont caractérisées par un syndrome opto-chiasmatique plus au moins sévère révélant la maladie dans 28,3% des cas [1]. Après l'atteinte du nerf optique, la paralysie d'un nerf crânien est le signe neurologique le plus fréquent notamment la 7^ème^ paire crânienne [9] suivie des nerfs occulomoteurs [6, 10, 11], des céphalées [12], ataxie, troubles cognitifs et un déficit neurologique [3, 4]. Des cas d'épilepsie et de névralgie faciale ont été aussi rapportés [6, 11]. Les signes sino-nasals ont été retrouvés dans 25% des cas dans cette localisation par rapport à 2% dans les autres localisations sur la série de Langrand [6]. Il a été constaté de même sur la série de 9 cas de sarcoïdose hypothalamo-hypophysairede Bihan *et al.* [8]. L'imagerie par résonance magnétique reste non spécifique malgré le caractère évocateur de la prise de contraste sellaire étendue à la tige pituitaire et plus généralement de la base du crâne [6]. Les anomalies les plus rencontrées en neurosarcoïdose est l'atteinte multiples de la substance blanche (43%) et la prise de contraste pathologique des méninges (38%) [11]. D'autres signes radiologiques peuvent être appréciés: une hydrocéphalie obstructive et l'atteinte de la moelle épinière. L'imagerie peut être normale dans 12% des cas [11]. La biologie est caractérisée par des troubles endocriniens, un taux de l'enzyme de conversion élevée (x 3,5) et le bilan doit être complété par la recherche de lésions systémiques. Les troubles endocriniens les plus rencontrés sont le diabète insipide et l'hyperprolactinémie et à moindre degré l'hypogonadisme [6, 8].

**Tableau 1:** Atteinte hypothalamo-hypophysaire dans le cadre d’une neurosarcoïdose; revue de littérature permettant de retrouver 9 cas d’atteinte isolé hypothalamo-hypophysaire de sarcoïdose

Etude	Année	Nombre de cas	Sexe Ratio F:H	Prise de contraste HH	Confirmation Histologique	Atteinte isolé hypothalamo-hypophysaire
**Anthony *et al* [1]**	2016	4	0:4	4	4/4	1/4
**Tabuena *et al* [5]**	2004	4	3:1	4	4/4	1/4
**Langrand *et al* [6]**	2012	24	10:14	14/24	21/24	4/24
**Bullmann *et al* [13]**	2000	5	2:3	4	-	2/5
**Prayson *et al* [14]**	2016	1	-	NP	1	1/1

(NP: non précisée)

Cependant sur une revue de littérature publiée par Anthony *et al.* [1], l'auteur a dressé un classement décroissant des troubles endocriniens: un hypogonadisme central (LH/FSH) dans 88,8% des cas, une hypothyroïdie centrale (TSH) dans 67,4% des cas, un diabète insipide dans 65,2% des cas, un taux de GH bas dans 54% des cas, un taux d'ACTH bas dans 48,8% des cas, une hyperprolactinémie dans 48,8% des cas. Sur la série de 68 patients de neurosarcoïdose rapporté par Zajicek *et al.* le taux de l'enzyme de conversion est retrouvé élevée chez 23,5% des cas seulement [11]. L'étude de l'enzyme de conversion n'a pas de grande valeur diagnostique de fait du faible taux de spécificité et de sensibilité ni de valeur pronostique pour juger de l'évolution des patients sous traitement médical [3]. L'analyse du Liquide céphalo-rachidien retrouve une hyperlymphocytose non spécifique [3, 6, 8]. L'étude multicentrique rétrospective publiée par Langrand *et al.* [6] a rapporté une série de 24 cas de neurosarcoïdose hypothalamo-hypophysaire. 22 patients avaient des troubles endocriniens dont 12 avec un diabète insipide. La prise de contraste sur l'IRM cérébrale a été notée chez les 14 patients. Il a été aussi rapporté que l'atteinte hypothalamo-hypophysaire s'accompagne de plus de signes neurologiques en comparaison avec les autres atteintes encéphaliques dans le cadre d'une neurosarcoïdose [6].

En 1999, Zajicek *et al.* ont décrit des critères cliniques et paracliniques pour le diagnostic d'une neurosarcoïdose selon 3 niveaux: certain, probable et possible [11]. Cette classification ne s'applique pas dans notre cas car le diagnostic est confirmé par anatomopathologie devant un cas de sarcoïdose hypothalamo-hypophysaire mimant un adénome hypophysaire. Ce cas rejoint les 09 cas déjà décrit sur la littérature de la localisation isolée hypothalamo-hypophysaire d'une sarcoïdose [1, 5, 6, 13, 14]. Le traitement est principalement médical avec administration des corticoïdes. La neurosarcoïdose se caractérise d'un taux de rémission après traitement médical de 8% alors qu'il est de 39% dans les autres localisations. Une amélioration radiologique est notée dans 29% à 78% des cas [6, 8]. L'insuffisance hypothalamo-hypophysaire est irréversible dans tous les cas malgré le traitement médical [5, 6, 8]. Un traitement hormonal substitutif est indispensable. D'autres thérapeutiques plus agressives peuvent être prescrites devant une mauvaise réponse à la corticothérapie ou l'agressivité de la manifestation de la neurosarcoïdose. Ces molécules sont principalement des immonosuppresseurs et des immunomodulateurs [1, 15, 16]: méthotrexate, mycophenolate mofetil, cyclosporine, azathioprine, l'infliximab et l'hydrochloroquine. Une radiothérapie peut être aussi indiquée dans certains cas réfractaires à l'ensemble des traitements médicaux possibles [16]. La prise en charge chirurgicale s'accompagne d'un taux élevé de fistule post opératoire de LCR et de diabète insipide avec des troubles endocriniens persistants malgré le traitement de la sarcoïdose avec la nécessité le plus souvent d'un traitement hormonal substitutif [6]. Un taux d'amélioration endocrinienne de 13% a été rapporté par Anthony *et al.* sur sa revue de littérature [1]. La rémission sous traitement est 5 fois moins favorable en cas de neuro-sarcoïdose [6]. Un cas de décès est rapporté sur la littérature suite à évolution fatale un an après le diagnostic d'une neurosarcoïdose suite à une méningo-encéphalite granulomateuse aigu [8]. Toutes causes confondues, le taux de mortalité est de 5% à 10% [1, 4].

## Conclusion

La neurosarcoïdose est un critère de mauvais pronostic chez un patient atteint d'une sarcoïdose. L'atteinte hypothalamo-hypophysaire est rare avec des complications plus fréquentes que les autres atteintes neurologiques et systémiques nécessitant une prise en charge multidisciplinaire à long terme.

## Conflits d’intérêts

Les auteurs ne déclarent aucun conflit d'intérêts.
